# Characterization of the complete chloroplast genome of *Gentiana rhodantha* (Gentianaceae)

**DOI:** 10.1080/23802359.2020.1718026

**Published:** 2020-01-27

**Authors:** Li-Zhen Ling

**Affiliations:** Key Laboratory for Specialty Agricultural Germplasm Resources Development and Utilization of Guizhou Province, Liupanshui Normal University, Liupanshui, China

**Keywords:** Chloroplast genome, *Gentiana rhodantha*, Gentianaceae, phylogenetic analysis

## Abstract

The first complete chloroplast genome (cp) sequences of *Gentiana rhodantha* were reported in this study. The cp genome of *G. rhodantha* was 148,967 bp in size, with two inverted repeat (IR) regions of 25,760 bp, the large single copy (LSC) region of 79,831 bp, and the small single copy (SSC) region of 17,616 bp. The cp genome contained 112 genes, including 78 protein-coding genes, 4 ribosomal RNA, and 30 transfer RNA genes. The overall GC content was 36.4%. Phylogenetic analysis of the cp genomes within the tribe Gentianeae suggests that *G. rhodantha* is in a sister clade of other subtribe Gentianinae.

*Gentiana rhodantha* Franch. ex Hemsl, is an annual herb of the family Gentianaceae and native to the southwest of China (Ho and Pringle 1995). The whole plant of *G. rhodantha* (commonly named Honghualongdan) is used as a traditional ethnomedicine for the treatment of hepatitis, jaundice, phthisis, and dysentery (Wu et al. 2011). Several researches have demonstrated that *G. rhodantha* is a rich source of iridoids and polyphenols and shows anti-inflammatory, hepatoprotective, and antimicrobial activities (Ma et al. 1994, 1996; Xu et al. 2008, 2011; Wu et al. 2011; Chen et al. 2013; Pan et al. 2015). In addition, mangiferin was confirmed as the characteristic compound to evaluate the quality of *G. rhodantha* (Wu et al. 2011). Here, we characterized the complete chloroplast (cp) genome of *G. rhodantha* based on the Illumina sequencing technology to understand the genetic background and explore its phylogenetic placement.

The specimen (lpssy0304) of *G. rhodantha* was collected from Longshan mountain, Liupanshui, China (N26°34′12″, E104°48′56″, 1,900 m) and deposited in the herbarium of the Liupanshui Normal University (LPSNU). The genomic DNA was extracted and used for sequencing as previously described (Zhang et al. 2019). About 2 Gb raw data were generated and used for *de novo* cp genome assembly with SPAdes (Bankevich et al. 2012) and all predicted genes were annotated using PGA (Qu et al. 2019).

The complete *G. rhodantha* cp genome (GenBank accession number: MN822304) is 148,967 bp in length, including a large single-copy (LSC) of 79,831 bp, a small single-copy (SSC) region of 17,616 bp, and a pair of inverted repeats (IRs) of 25,760 bp each. The cp genome shows the GC content of 37.7% and contains 112 unique genes, including 78 protein-coding genes, 30 transfer RNA (tRNA) genes, and 4 ribosomal RNA (rRNA) genes. Among them, 14 distinct genes (*atpF*, *ndhA*, *ndhB*, *petB*, *petD*, *rpl16*, *rpl2*, *rpoC1*, *trnA-UGC*, *trnG-UCC*, *trnI-GAU*, *trnK-UUU*, *trnL-UAA*, and *trnV-UAC*) contain one intron and three genes (*clpP*, *rps12* and *ycf3*) have two introns.

The family Gentianaceae is in the major group Angiosperms and used in medicine as well as in gardening. Gentianaceae contains six tribes and over 1600 species (Sun and Fu 2019). Among them, the tribe Gentianeae comprises approximately 940 species and is classified into two subtribes: subtribe Gentianinae and Swertiinae (Sun and Fu 2019). To determine the phylogenetic position of *G. rhodantha* within Gentianeae, we obtained the complete cp genome sequence data from 20 species of Gentianeae in GenBank (Figure 1). Sixteen species from Apocynaceae were used as outgroups in this analysis. In this study, the sequence dataset was aligned automatically using MAFFT version 7.0 (Katoh and Standley 2013) with manual corrections. We inferred maximum likelihood (ML) and Bayesian inference (BI) (Ronquist et al. 2012; Stamatakis 2014) trees from the dataset, which generated the same tree topology (Figure 1). A framework of the phylogeny with support for two subtribes was obtained. The phylogenetic analysis showed that *G. rhodantha* was in a sister clade of other subtribe Gentianinae (Figure 1).

**Figure 1.**
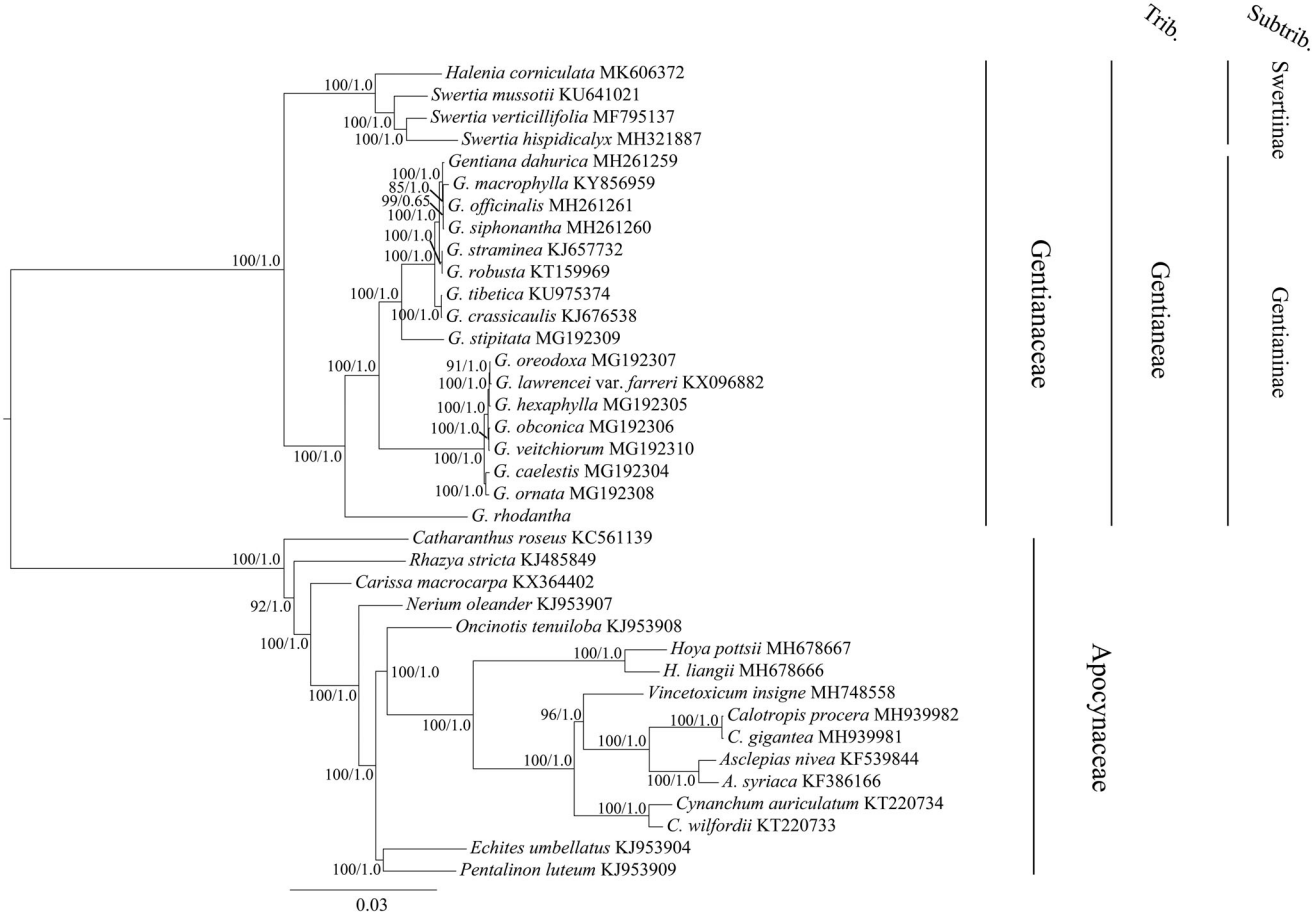
The maximum likelihood (ML) tree of Gentianeae inferred from the complete chloroplast genome sequences. Numbers at nodes correspond to ML bootstrap percentages (1,000 replicates) and Bayesian inference (BI) posterior probabilities.
